# The hidden mental health burden of corruption in healthcare: Findings from a scoping review and key informant interviews

**DOI:** 10.1371/journal.pgph.0007509

**Published:** 2026-10-30

**Authors:** Madura Nanthakumaran, Charles Orjiakor, Dina Balabanova

**Affiliations:** 1 Department of Public Health, Environments and Society, London School of Hygiene and Tropical Medicine, London, United Kingdom; 2 Department of Psychology, University of Nigeria, Nsukka, Nigeria; 3 Department of Global Health and Development, London School of Hygiene and Tropical Medicine, London, United Kingdom; Universiti Kuala Lumpur Royal College of Medicine Perak, MALAYSIA

## Abstract

Corruption in healthcare is a pervasive global challenge that undermines equity, efficiency, and trust in health systems. Whilst its economic and governance impacts are well documented, the psychological consequences for patients, providers, and those involved in anti-corruption reform remain under-explored. Understanding these mental health effects is critical for designing anti-corruption strategies that safeguard wellbeing and strengthen health systems. An exploratory mixed-methods study was conducted to map an emerging evidence base. A scoping review of 25 peer-reviewed and grey literature sources was complemented by four key informant interviews, which were used to interpret and contextualise the review findings. Findings were synthesised into a conceptual framework mapping pathways between corruption and mental health outcomes. Corruption was reported to be associated with psychological distress among patients, providers, and anti-corruption actors. This included stress, anxiety, depressive symptoms, and moral injury. At a community level, corruption eroded trust and collective agency, whilst institutional effects included normalisation of unethical practices. Structural determinants were found to perpetuate corruption’s harms. Anti-corruption efforts produced mixed outcomes: reform targeting high-level officials improved public mental health modestly, whereas punitive measures against frontline workers exacerbated fear and demoralisation in the workforce. Protective factors such as moral agency, and robust reporting systems were found to buffer harm but were context dependent. Policy recommendations are made for practice, research and capacity building. These include framing corruption as a public mental health issue, strengthening whistleblower protections, commissioning further research, and adapting reform to local political economy and social norms. These findings indicate that corruption is a plausible but under-recognised social determinant of mental health requiring further research. Integrating mental health considerations into anti-corruption policy and health system reforms is essential to mitigate harm and strengthen resilience.

## Introduction

Corruption does not have a single general or universal definition, and different scholarly, policy, and institutional traditions emphasise different aspects of the concept. In this study, we use Transparency International’s widely cited definition which is also close to definitions used by multilateral agencies: “the abuse of entrusted power for private gain”. This is useful for mapping mechanisms across heterogeneous settings, while recognising that other definitions may place greater emphasis on informal authority, institutional practices, or context-specific social obligations. Corruption can manifest across the health system, from frontline service delivery to high-level policy and administration [1,2]. The United Nations General Assembly explicitly recognised corruption as a barrier to resource mobilisation and allocation, undermining efforts to achieve universal health coverage (UHC) [3], defined as the principle that individuals should be able to access essential health services without financial hardship. Structural vulnerabilities in the health sector, including information asymmetry between providers and patients, uncertainty in demand, and multiple stakeholders, make it particularly susceptible to corruption [4].

Corruption in healthcare can be understood using four interrelated dimensions [4]. The first involves bribes and informal payments, where patients are required to pay unofficial fees to receive or expedite care. The second is theft and embezzlement, referring to the misappropriation of resources or funds intended for public use. Fraud and falsification include manipulation of records, billing for services not provided, and procurement fraud. Lastly, favouritism and nepotism manifest when employment, promotions, or access to care are based on connections rather than merit or need. The conditions that allow corruption to take root are known as the “fraud triangle.” This includes opportunity, arising in environments with weak oversight; pressure, such as low salaries that push individuals toward unethical behaviour; and rationalisation, where corruption becomes morally justified [4]. At a systemic level, corruption exacerbates inequities, undermining institutional legitimacy and public trust. This leads to reduced service utilisation and poorer health outcomes.

The definition outlined above rests on a particular understanding of corruption, which is contested. It centres discrete acts committed by individuals holding formal office, and in doing so privileges certain framings of the problem. Corruption in the health sector spans a broad range of practices, including unethical behaviour through administrative misdemeanour, to criminal offences [5]. Whilst there is broad cross-national agreement about the latter, practices such as informal payments and absenteeism require context-specific interpretation [5]. Alternative definitions foreground who holds power over resources and how informal authority competes and interacts with formal institutions [6]. A related distinction is drawn between individual corruption, which largely comprises criminal conduct, and institutional corruption, where behaviour forming an accepted or even required part of institutional practice nonetheless diverts an institution from its social purpose [7]. These choices are consequential, as the assumptions made about what constitutes corruption shape which responses are considered and its harms [8,9]. This study takes the Transparency International definition as its reference point, whilst recognising that included sources and key informants may apply broader or differently grounded understandings. This is consistent with a growing body of work framing corruption as a structural rather than individual phenomenon [7,10].

Recent reviews have emphasised the harms of health sector corruption. Corruption presents a fundamental threat to achieving the Sustainable Development Goals (SDGs), particularly SDG 3 (health) and SDG 16 (justice and strong institutions) [11]. Systemically, corruption deepens inequities and erodes institutional legitimacy, leading to lower service use and poorer health outcomes. As a result, policy discourse has begun to frame corruption as a global crisis requiring coordinated, evidence-based reform. This moves beyond addressing visible individual acts, to interventions which address the deeper structural factors which sustain corruption [10,12].

Efforts to address health sector corruption have developed in recent years. In 2000, the United Nations Committee on Economic, Social and Cultural Rights (CESCR) adopted General Comment Number 14, interpreting the right to the highest attainable standard of physical and mental health and treating misallocation of public resources that results in non-enjoyment of this right as a violation of state obligations [13]. This, together with the UN Convention against Corruption in 2003 [14], helped establish the normative and legal foundations for anti-corruption efforts. Following this, Vian’s analytical framework and guidance from organisations such as the former United Kingdom Department for International Development (UK DfID), WHO, the U4 Anti-Corruption Resource Centre, and Transparency International, conceptualised corruption as a structural issue [2,4,15–18]. In 2015, SDG 16.5 called for reductions in bribery and corruption in all forms, and this priority was linked to the broader context of health systems strengthening in WHO’s Anti-Corruption, Transparency and Accountability framework [11,16]. More recently, anti-corruption has been integrated into global health governance. The Global Network on Anti-Corruption, Transparency and Accountability (GNACTA), which was established in 2019, works to unite governments, donor organisations and civil society to promote accountability and evidence-based reform [19]. In 2021, the United Nations Office on Drugs and Crime (UNODC), which supports implementation of the United Nations Convention against Corruption [14], published guidelines for whistleblower protection in the health sector [20]. These guidelines aim to create safe environments and protect those speaking out against corruption, with some elements recognised as promoting psychological safety. Most recently, the Lancet Global Health commission on anti-corruption in health has called for a novel approach that goes beyond existing efforts [21].

Corruption’s harms also engage binding obligations. The International Covenant on Economic, Social and Cultural Rights obliges states to realise the right to the highest attainable standard of physical and mental health to the maximum of their available resources [22], and misallocation of public resources through corruption therefore bears on the right to health systemically [13,23,24]. Therefore, it is important to understand how corruption affects the right to health, including the mental health consequences for those who encounter it or seek to reform it.

Although current anti-corruption efforts emphasise institutional reform, they primarily focus on financial oversight and legal enforcement. Emerging evidence demonstrates that corruption has a measurable impact on population health, such as reduced access to healthcare and increased mortality [21]. However, little is known about the association between corruption and mental health [25,26]. A few studies across geographical contexts have demonstrated the harmful effects of corruption to include increased anxiety, depression, moral injury, and diminished trust [26–29], but the scale of these impacts has not been considered.

Understanding the mental health impacts of corruption is important, as this can influence how people view and utilise healthcare, as well as willingness to engage in collective action. Therefore, this study aims to take a global health systems perspective to examine how corruption in healthcare affects mental health and psychosocial wellbeing. A scoping review of and key informant interviews are used to explore how corruption is experienced by healthcare users, healthcare providers, and those engaged in anti-corruption reform. Finally, a conceptual framework is proposed to map these pathways, and recommendations are made to address the mental health outcomes of corruption and anti-corruption reform.

## Methods

### Ethics statement

Ethical approval was granted by the London School of Hygiene and Tropical Medicine MSc Research Ethics Committee on 27 May 2025 (LEO-CARE Reference: 31966). Written informed consent was obtained from all participants prior to interview, and verbal consent was additionally recorded at the start of each interview.

### Overview of methods

This exploratory mixed-methods study combined a scoping review with semi-structured key informant interviews. The scoping review provided the primary evidence base, and the interviews were conducted subsequently to interpret, contextualise, and identify gaps in the review findings. Evidence from both components was then integrated to develop the conceptual framework.

### Scoping review

A scoping review was conducted to examine how corruption in healthcare and public sector institutions affects mental health and psychosocial wellbeing. This type of review was chosen due to the limited literature on corruption’s mental health impacts; as such, the scope was expanded to include evidence from other public sectors, where findings offered transferable insights. The review followed the methodological framework of Arksey and O’Malley and the Preferred Reporting Items for Systematic reviews and Meta-Analyses extension for Scoping Reviews (PRISMA-ScR) checklist guided its reporting (S1 File), [30,31]. No protocol was registered or published in advance of this review.

#### Search strategy.

Database searches were conducted in June 2025 across MEDLINE, Global Health, APA PsycINFO, and Scopus. Search strategies combined three core concepts: corruption and governance, mental health and psychosocial outcomes, and relevant stakeholders or organisations.

Grey literature was identified through Google searches and targeted queries of WHO and UN websites using the same conceptual structure. The first 50 items were screened, as relevance declined beyond this point. Browser cookies were cleared to minimise bias. Full search strategies are provided in the supplementary material (S1 Text). Additional articles were identified through expert recommendation in interviews, and reference screening.

Sources were included if they examined mental health or psychosocial impacts of corruption or anti‑corruption at a population or individual level. Eligible sources included primary studies, reviews, policy reports, and analytically grounded conceptual papers addressing the experiences of patients, service users, healthcare workers, whistleblowers, reformers, or public officials. Evidence from other public sector settings was included where the mechanisms of corruption and its psychosocial effects were judged to be comparable to healthcare. Only English-language sources published between 2000 and June 2025 were considered. This period was chosen to reflect the emergence of rights-based and anti-corruption frameworks and recent literature relevant to healthcare, as outlined in the Introduction.

Sources were excluded if they only focused on financial, legal, or institutional dimensions of corruption, or if full texts were not available.

#### Screening.

All results were exported to reference manager software. Duplicates were removed, and one reviewer (MN) screened titles, abstracts, and full texts against criteria. Reference list snowballing identified additional papers.

#### Data extraction.

A structured data extraction sheet was developed to include publication details, setting, design, aims, population or group of interest, context of corruption, and reported mental health outcomes. For primary studies, sample size and methods of outcome assessment were recorded. Data extraction was conducted by one reviewer (MN) and inclusion discussed with other authors. No formal risk‑of‑bias assessment of included studies was undertaken, as this was a scoping review aiming to map the breadth and nature of available evidence.

#### Data analysis and synthesis.

MN conducted the initial analysis of included papers and reports, summarising key findings and interpretations. This was shared with co‑authors, and differences in interpretation were discussed for consistency and clarity in the synthesis. Data analysis followed a framework analysis approach, to allow a flexible approach to synthesise evidence given the relative absence of a theoretical model explicitly connecting corruption to mental health. Framework analysis enabled the combination of deductive structure with inductive sub-theme development [32,33].

A deductive framework was developed in advance based on preliminary scoping of literature and discussions with experts in health systems corruption and mental health. It incorporated a systems and social determinants of health approach, recognising that impacts extend beyond the individual [34]. This framework consisted of predefined themes. The final two themes were used to map specific pathways from corruption to mental health outcomes and support development of the conceptual framework:

1. Negative individual psychological impacts of corruption
2. Negative social and community-level impacts of corruption
3. Structural determinants of corruption; formal and informal systems
4. Protective factors for mental and psychosocial wellbeing
5. Positive and negative mental health and psychosocial impacts of anti-corruption
6. Pathways linking corruption and anti-corruption to psychosocial and mental health outcomes
7. Systems-level interactions between corruption and mental health, considering individual, community, institutional, and structural factors.

During full‑text review, inductive coding was used to identify sub‑themes that emerged within each of these predefined domains. This approach enabled flexibility while ensuring that coding remained grounded in the core areas of interest. Evidence from healthcare and non-healthcare settings was compared to assess if findings converged or diverged. Findings were synthesised narratively to describe how corruption influences mental health, given the heterogeneity of study designs, outcomes, and contexts.

### Key informant interviews

To help contextualise findings from the literature, semi-structured interviews were conducted with experts in health policy, governance, and anti-corruption in healthcare. Participants were purposively sampled to include experts based on their experience in health systems research or senior roles in anti-corruption reform. Participants were identified through authorship of key peer-reviewed publications that explore association between corruption and mental health, and snowball sampling was used via professional networks.

Fifteen individuals were invited to participate by email between July 2025 and August 2025. Four individuals declined due to limited availability or uncertainty about the relevance of their expertise. The remaining non‑responders did not reply despite two reminders. Four agreed to take part and were interviewed as key informants by MN, who has had previous experience in conducting focus groups and semi-structured interviews.

#### Data collection.

Interviews were conducted using Zoom between July 2025 and August 2025 by MN. A semi-structured topic guide (S2 Text) explored five domains:

1. Experiences and manifestations of corruption in healthcare
2. Mental health and psychosocial impacts on stakeholders
3. Structural influences and enabling conditions
4. Individual and institutional coping strategies
5. Policy and system-level responses

The guide was used flexibly to reflect participant expertise, and to enable the identification of new subthemes within these domains where participants elaborated on issues not captured in the literature. Participants received an information sheet and provided written and verbal informed consent prior to the interview (S3 Text), these were stored securely. Interviews were audio-recorded and transcribed verbatim.

#### Data analysis.

Transcripts were anonymised and analysed using framework analysis in NVivo 15 by MN. The same thematic structure used in the scoping review was applied deductively to derive common themes, and inductive sub-coding was used to capture new insights and differences of opinion. Data was summarised in a matrix to support comparison and narrative synthesis, and findings shared with co-authors for discussion.

### Conceptual framework

The conceptual framework was developed iteratively through triangulation of findings from the scoping review and interviews. An initial exploratory figure helped map broad themes and subthemes, for example, low mood and anxiety as individual psychological impacts resulting from corruption. This was gradually refined as analysis progressed, to visualise pathways through which corruption and anti-corruption efforts affect mental health, including mediating and protective factors. The final figure derived after the completion of the analysis is presented in the Discussion.

## Results

### Scoping review

Database searches yielded 509 peer-reviewed articles. Six additional articles were identified through expert recommendation, and four through reference screening. Grey literature searches returned 53 sources, and nine through snowballing and expert recommendation. The final sample included 25 papers and reports: 19 peer-reviewed and six grey literature papers. A flow diagram of article selection is presented in Fig 1.

**Fig 1:**
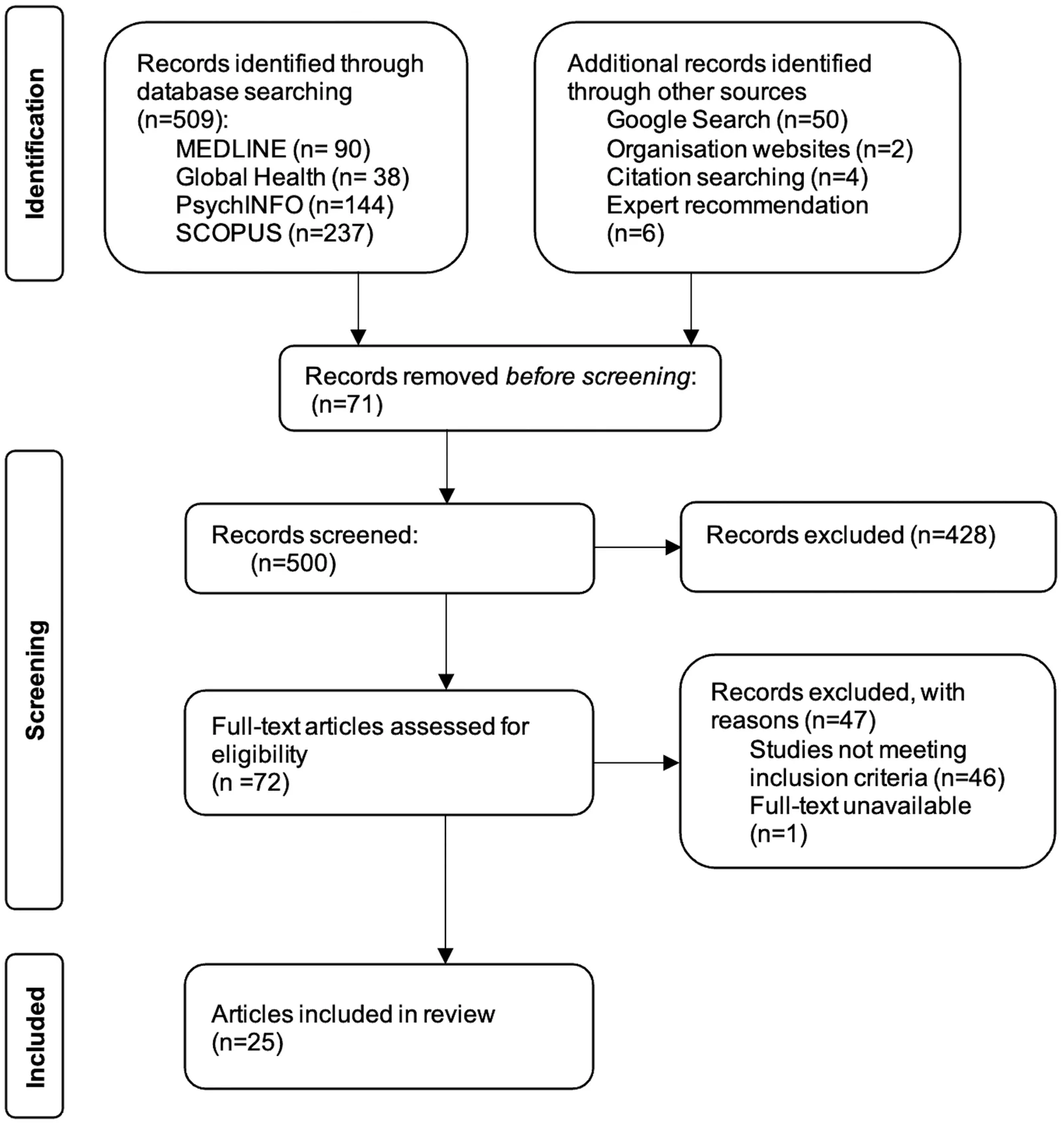
Scoping review flow diagram for selected studies.

#### Descriptive analysis.

Most included studies were published between 2018 and 2024, reflecting recent attention to corruption’s effect on mental health. Methodologies included qualitative (n = 7), quantitative (n = 8), mixed methods (n = 5), and reviews (n = 4). The populations studied included healthcare workers, patients, civil servants, whistleblowers, and the public. Mental health outcomes were assessed through validated instruments and qualitative or survey methods. While several studies focused on healthcare, others from broader public sectors offered useful insights with relevance to health. Larger studies were population-level analyses spanning multiple public sectors and relied upon measures of perceived corruption, whereas healthcare-specific evidence derived predominantly from small qualitative studies. No included source combined a healthcare-specific focus with large-scale or representative sampling.

Definitions and operationalisation of corruption varied substantially across included sources. Six studies measured perceived corruption using survey items or composite indices at individual or country level [26,35–39], and one examined public perceptions of governmental actions during crisis response [40]. Four operationalised corruption as informal or unofficial payments for care [29,41–43], and two as specific service-delivery failures, namely absenteeism and bribery in primary care [28,44]. Three treated corruption as an organisational or psychological process, focusing on normalisation, tolerance and moral desensitisation rather than discrete transactions [45–48], and two related papers measured bribe-offering as a behavioural outcome in experimental message testing [49,50]. Single studies examined self-reported engagement in corrupt practice among public officials [51], retaliation following disclosure of wrongdoing [52], governance failure in resource allocation [53], and institutionally driven exclusion from care [54]. The remaining grey literature applied policy typologies of corruption risk across health system functions [20,55]. No single definition was shared across sources, and the review therefore maps a family of related phenomena rather than a single consistently defined exposure. Results are summarised in Table 1.

**Table 1:** Summary of included studies in the scoping review.

Report	Setting	Method	Aims	Corruption Setting	Population examined (sample size is included for primary research)	Key Findings / Recommendations
**Anyan et al. (2024)** [26**]**	Ghana	Quantitative	Examine how perceptions of corruption are associated with mental health problems controlling for trust and socio-cultural factors.	Population-level (multi-sector public institutions)	Ghanaian population in tertiary level education (N = 730)	Perceived corruption in political and institutional systems most strongly associated with depression, followed by anxiety. Urban residents, women, and individuals with moderate religiosity perceived higher corruption.
**Ahmed et al. (2024)** [53**]**	Pakistan	Mixed methods	Investigate experiences of resource insecurity and malnutrition in Southern Punjab.	Cross-sector public services (water, nutrition, healthcare)	General population from water insecure areas (n = 235; survey data, n = 10; qualitative), community members identified as having knowledge of water insecure areas, history and geography (n = 5), and healthcare providers (n = 20)	Governance failures and corruption in water and nutrition sectors hinder access and equity. Higher perceived stress associated with water and food insecurity.
**Keilman et al. (2023)** [54**]**	United States	Qualitative	Explore how nurses experience moral distress when patients forgo care due to cost.	Healthcare	Nurses who had been in practice for at least one year from various healthcare settings (N = 19).	Nurses commonly encountered patients foregoing treatment and reported moderate moral distress. Findings indicate systemic injustice and providers employed informal strategies to support patients, which led to stress and frustration.
**Wright et al. (2022)** [40**]**	United Kingdom	Cross-sectional Qualitative	Explore public perceptions of government performance during COVID-19 and its impacts on mental wellbeing and trust.	National governance / political system	General adult population in UK. Additional targeted sampling of populations with low income, no or few educational qualifications, unemployment and engaged with third sector organisations for vulnerable groups (N = 4054).	Themes included mistrust, leadership failure, inconsistent messaging, and perceived corruption (e.g., cronyism, opaque contracting). Associated outcomes included disillusionment, helplessness, and reduced trust.
**van der Velden et al. (2019)** [52**]**	Netherlands	Quantitative	Assess prevalence and severity of mental health problems among whistleblowers compared to general population and at-risk groups	Cross-sector: public (healthcare, civil service) and private sectors.	Whistleblowers identified from anonymous register. Matched controls included general population. With comparators of healthy individuals, work-disabled persons and patients with cancer.	Whistleblowers showed significantly higher rates of anxiety, depression, distrust, agoraphobia, and sleep disturbances. Psychological impacts were comparable to those with serious physical illness.
**George et al. (2018)** [41**]**	Romania	Qualitative	Explore perceived barriers to accessing healthcare among a vulnerable community and if these reflect the health care access barriers model	Healthcare	Adults living within or working with a vulnerable community residing in an area of East Romania (N = 18).	Corruption emerged as a financial barrier in nearly all accounts. Psychological barriers and impacts of reduced access emerged as a discrete theme associated with hopelessness, fear and anxiety.
**van Deurzen (2017)** [35**]**	Europe	Quantitative	Examine whether perceived corruption is associated with mental health, and affects groups more vulnerable to corruption (material adversity, women, religion, former communist states)	Population-level (multi-sector public institutions)	General population from 24 countries in European Social Survey (2006, 2012, 2014; N = 99,159)	Higher perceived corruption was associated with greater depressive symptoms in static models. Depressive symptoms higher in those reporting greater material adversity and higher religiosity.
**Rahmani et al. (2013)** [42**]**	Afghanistan	Qualitative	Explore experiences of antenatal and obstetric care provision in Afghanistan	Healthcare	Service users of antenatal and obstetric clinics and healthcare providers in Kabul and Ghazni (N = 27)	Antenatal care was underutilised despite availability. Barriers included financial constraints, limited awareness, and corruption (bribery, favouritism, lack of accountability). Emotional impacts included fear, humiliation, and helplessness.
**Vian et al. (2006)** [29**]**	Albania	Qualitative	To identify drivers and perceptions of informal payments in healthcare	Healthcare	General public and healthcare providers across health facilities in three Albanian districts (N = 131).	Informal payments driven by low salaries, weak governance, and cultural norms. Perceived costs included stress, reduced trust, and compromised care. Benefits included perceived attentiveness and quality care.
**Achim et al. (2020)** [36**]**	Global	Mixed methods	To examine the relationship between corruption and health and whether these relationships differ between HIC and LICs.	Population-level (multi-sector public institutions)	Country-level analysis using data from 2005-2017 (N = 185; 54 HICs and 131 LICs)	Corruption was associated with increased under 5 mortality and lower life expectancy, with the effect more pronounced in LICs. Perceived corruption reduced happiness overall, but not in LICs. Collectivism protected health in LICs; individualism increased happiness in HICs.
**Fleming et al. (2022)** [45**]**	Greece	Qualitative	Examine persistence of corruption and its normalisation	Cross-sector: public (healthcare, civil service) and private sectors.	Mid- to late-career managers in the public sector, or equivalent managers in private sector who deal extensively with public sector organisations (N = 58).	Corruption widely acknowledged in public institutions. Persistence explained by “second-order normalisation,” where tolerance among uninvolved actors perpetuates corruption.
**Garret et al. (2016)** [46**]**	N/A	Experimental study	Investigate whether dishonesty escalates over time and underlying neural mechanisms	Experimental/individual-level	Adult volunteers from UK-based psychology subject pool. (N = 58)	Repeated dishonest acts for personal gain led to increasing dishonesty and reduced amygdala activation. Supports a “slippery slope” neurological model of moral desensitisation.
**Ashforth et al. (2003)** [47**]**	N/A	Theoretical model development and literature review	Create a conceptual model to explain how corruption becomes normalised in organisations	Conceptual/organisational	N/A	Corruption becomes embedded through institutionalisation, rationalisation, and socialisation. These processes reduce ethical sensitivity and integrate corruption into organisational norms.
**Cheeseman et al. (2022)** [49**]**	Nigeria	Quantitative	Examine effects of anti-corruption messages on bribery and perceived corruption	Population-level (multi-sector public institutions)	General population in Lagos (N = 2572)	Most anti-corruption messages increased bribe-offering. A tax-framed message reduced bribery among less pessimistic individuals. Content and framing shaped behavioural outcomes.
**Sharma et al. (2021)** [37**]**	Vietnam	Quantitative	Assess relationship between corruption and mental health, and impact of an anti-corruption campaign	Population-level (multi-sector public institutions)	General population residing in rural communes and rural household enterprises	Strong evidence to suggest that corruption is associated with depressive symptoms. The 2016 anti-corruption campaign led to improvement in depressive symptoms in areas with higher baseline corruption.
**Fu et al.** **(2024)** [38**]**	China	Quantitative	Assess the mental health effects of anti-corruption campaigns	National governance/political system	General population and households in China, with subgroup analyses by government employment status	Government employees had increased depressive symptoms post-campaign; ordinary citizens did not have a change. Perceived corruption declined in both groups.
**Orjiakor et al. (2023)** [28**]**	Nigeria	Qualitative	Explore narratives on how staff absenteeism is rationalised or resisted by providers and service users.	Healthcare	Healthcare providers, managers and councillors in primary health centres (n = 34) and service users (n = 22)	Absenteeism was widespread and normalised. Political protection and community ties shielded staff from consequences. Feelings of frustration and hopelessness were common among staff and service users.
**Peiffer et al. (2021)** [44**]**	Uganda	Mixed methods	Investigate why bribery reduced in health sector	Healthcare	Health service providers, service users, and sector experts from Uganda’s Central, Eastern, and Western provinces.	The Health Monitoring Unit helped reduce bribery. Unintended effects were low staff morale and trust.
**DeSouza et al. (2025)** [51**]**	Ghana	Mixed methods	How engagement in corruption affects public sector workers’ mental well‑being and what factors explain this.	Public sector (non-health)	Public sector workers involved in corrupt practices	Negative effects were low mood, stress, anxiety, sleep problems and low self-esteem. Moderating factors included positive social standing, power, lack of moral conflict and religion.
**Cuadrado (2020) [27]**	Global	Policy report and case review	Analyse corruption risks in health service delivery during COVID-19 and threats to universal health coverage	Healthcare	Patients, frontline healthcare workers, and vulnerable population groups	Advised integrating anti-corruption into system reforms. Emphasised prevention, context-alignment, and civil society’s role in advocacy, documentation, and local accountability.
**UNODC** **(2021)** [20**]**	N/A	International best-practice guidelines	Recommend effective whistle-blower protections	Healthcare	Healthcare workers, patients, regulatory bodies, and legal and professional advisors.	Guidance included protected reporting channels, confidentiality, sanctions against retaliation, support mechanisms, and feedback structures.
**Gillanders (2011)** [39**]**	Sub-Saharan Africa	Quantitative	Examine mental health impacts of petty corruption in public services	Population-level (multi-sector public institutions)	General population of 18 countries completing Afrobarometer survey rounds 2 and 4 (2002/3 and 2005/6, N = 26,513)	Countries with higher corruption reported poorer mental health, after controlling for demographic variables.
**Hoffmann et al. (2017)** [48**]**	Nigeria	Mixed methods	Examining how social norms shape and sustain corruption in Nigeria, and how these inform anti-corruption strategies.	Population-level (multi-sector public institutions)	General population, anti-corruption practitioners, and governments officials	Entrenched norms and institutional weakness enabled corruption. Recommended behavioural insights into anti-corruption, streamlining processes, reducing incentives, and empowering leaders in communities to reshape expectations and promote anti-corruption.
**Peiffer et al. (2023)** [50**]**	N/A	Policy report and literature review	Summarise the effects of anti-corruption awareness campaigns.	Experimental/individual-level	Review of 9 experimental studies testing 19 messages in 7 countries	Most messaging had no impact or backfired. Descriptive norm messaging was harmful. Advised rigorous pre-testing before deploying such campaigns.
**Besa Global (2025)** [43**]**	Nigeria	Qualitative	Examine how gender, faith, and social norms intersect to normalise informal payments in healthcare	Healthcare	Healthcare providers and service users	Corruption is driven by material shortages, low salaries, and deeply embedded social, gender, and faith norms that pressure both health workers and service users to comply. It reinforces inequality and undermines trust. Multiple intersecting norms sustain corruption. Stakeholders are navigating a system which has negative psychosocial consequences such as chronic stress.

Included sources spanned Sub-Saharan Africa (n = 8), Europe (n = 6), Asia (n = 4) and North America (n = 1), alongside two global analyses and four conceptual or experimental sources reporting no geographic setting Table 2. Nigeria accounted for four sources, and no evidence was identified from Latin America, the Caribbean, the Middle East or Oceania. Patterns differed by income setting more than by region. In Low- and middle-income countries (LMICs), corruption was most often examined as informal payments, absenteeism and patronage within service delivery, whereas in high income countries (HICs) it appeared in more structural forms, including commodification of care and opaque contracting. Achim et al., was the only source designed to compare perceived corruption and mental health outcomes between LMICs and high income countries (HICs) [36].

**Table 2:** Distribution of included sources by region, with forms of corruption examined and reported psychological outcomes.

Region (n)	Included sources	Predominant forms of corruption examined	Reported mental health and psychosocial outcomes	Observations
Sub-Saharan Africa (n = 8)	Anyan et al. (Ghana); DeSouza et al. (Ghana); Cheeseman et al. (Nigeria); Orjiakor et al. (Nigeria); Hoffmann et al. (Nigeria); Besa Global (Nigeria); Peiffer et al. 2021 (Uganda); Gillanders (18 countries)	Perceived corruption at population level; informal payments in healthcare; absenteeism; bribery; engagement in corrupt practice by public sector workers; anti-corruption messaging and enforcement	Depression and anxiety associated with perceived corruption; poorer population mental health at country level; frustration and hopelessness among providers and service users; chronic stress; low mood, sleep problems and diminished self-esteem among those engaged in corruption; reduced morale and trust following enforcement	Largest regional grouping, with four sources from Nigeria. Most evidence on social, gender and faith norms sustaining informal payments, and on psychological costs in frontline workers.
Europe (n = 6)	van Deurzen (24 countries); Vian et al. (Albania); George et al. (Romania); Fleming et al. (Greece); Wright et al. (United Kingdom); van der Velden et al. (Netherlands)	Perceived corruption at population level; informal payments; corruption as a barrier to access; organisational normalisation; cronyism and opaque contracting during COVID-19; retaliation following disclosure	Depressive symptoms, stress and reduced trust, hopelessness, fear and anxiety, disillusionment; for whistleblowers, depression, and sleep disturbance, comparable in severity to serious physical illness	Most heterogeneous grouping, spanning transition economies and high-income settings. The only region contributing quantitative evidence on whistleblower outcomes.
Asia (n = 4)	Ahmed et al. (Pakistan); Rahmani et al. (Afghanistan); Sharma et al. (Vietnam); Fu et al. (China)	Governance failure and corporate influence across public services; bribery and favouritism in obstetric care; petty corruption; national anti-corruption campaigns	Perceived stress associated with resource insecurity, fear, helplessness, depressive symptoms. These improve following an anti-corruption campaign in high-corruption areas. Increased depressive symptoms among government employees following a campaign targeting lower-ranking officials	The only grouping containing evaluations of anti-corruption campaigns with population mental health outcomes. Findings diverge according to whom enforcement targets.
North America (n = 1)	Keilman et al. (United States)	Institutionally driven exclusion from care through insurance cost	Moral distress, stress and frustration among nurses encountering patients foregoing treatment	Single source. Corruption operationalised as institutional rather than individual, extending beyond definitions requiring private gain by an officeholder.
Global or multi-region (n = 2)	Achim et al. (185 countries); Cuadrado / Transparency International	Country-level corruption indices; corruption risks across health service delivery during COVID-19	Reduced happiness overall but not in low-income countries; collectivism protective of health in low-income countries, individualism associated with greater happiness in high-income countries	Achim et al. is the only included source designed to compare high- and low-income settings directly.
No geographic setting (n = 4)	Ashforth and Anand; Garrett et al.; UNODC; Peiffer et al. 2023	Conceptual and experimental accounts of normalisation, rationalisation and escalating dishonesty. International guidance on protection for reporting persons; review of anti-corruption messaging	Reduced ethical sensitivity and moral desensitisation; escalation of transgression following repeated dishonest acts	Conceptual, experimental or guidance sources without a defined setting. These contribute mechanism rather than context-specific evidence.

*Cross-national analyses are classified by the region they cover. Most settings are represented by a single source, and findings should not be interpreted as characterising regions. HIC, high-income country; LIC, low-income country; LMIC, low- and middle-income country.*

#### Individual psychological impacts of corruption on mental health.

Across included sources, the most consistent finding was the negative emotional toll of corruption on individuals. Exposure to informal payments or service denial due to corruption was associated with stress, hopelessness, anxiety, and depressive symptoms [26–29,35,37,39,43]. In LMICs, women, low-income individuals, rural populations, and ethnic minorities were more vulnerable to harm, due to higher exposure, and powerlessness in challenging corrupt systems [27,29,41–43,51,53]. This contributed to a loss of agency, heightening perceptions of hopelessness and anxiety.

Corruption affected victims and perpetrators. Workers engaged in systemic corruption reported restlessness, insomnia, low mood, and diminished self-esteem, often associated with workplace fear [43,51]. Among healthcare providers, particularly those operating in fragile health systems, corruption-related distress was associated with eroded professional identity and moral conflict [28,29,42]. In HICs, corruption manifested in structural forms, such as the commodification of care. In the United States, nurses described emotional strain, which they linked to institutional policies that constrained their ability to act ethically or uphold professional values [54]. This aligned with the concept of moral injury seen across studies [28,42,54].

Gender was found to moderate the effect of corruption on mental health. Ahmed et al. documented high levels of psychological distress among females navigating corrupt water and sanitation systems in Pakistan. The psychosocial burden of repeated exclusion, harassment, and informal gatekeeping left many experiencing chronic emotional strain, reporting access to some support programmes as exhausting and frustrating [53]. Several studies identified gendered differences in how corruption is experienced and sanctioned. In Nigeria, women who participated in corruption were judged more harshly [48]. Women therefore faced both institutional discrimination and moral stigma. Men described a loss of self-worth when unable to make informal payments for healthcare, and both risked blame if they could not secure care for dependents [43]. Religious belief also emerged as a moderating factor. In Ghana and Europe, higher religiosity intensified depressive symptoms associated with corruption [26,35]. Both papers hypothesised that highly religious individuals may experience greater emotional distress in response to corruption, likely due to increased moral dissonance.

#### Social and community-level psychological impact.

Corruption eroded trust, leading to collective disengagement and psychological distress [27–29,35,40,41,43,45,48,53,54]. This reflected shared psychosocial responses to fear and perceived futility, with marginalised groups experiencing heightened effects due to discrimination and barriers to access [41,53].

Across settings, trust in institutions was undermined. In the UK, opacity and inconsistency in COVID-19 policies, coupled with corruption during crisis response, created a collective sense of betrayal and delegitimised national institutions [40]. Similarly, in Vietnam, regression analysis showed that increased exposure to corruption was associated with a decrease in interpersonal trust and institutional trust [37].

Corruption also weakened civic engagement. In Pakistan, poorer communities facing corruption in food and water provision reported withdrawal and disempowerment after navigating bureaucratic hurdles, particularly marginalised groups [53]. In Albania, opaque informal payment systems fostered reliance on social networks and informal brokers [29]. In Ghana, systemic corruption within the public sector created community mistrust, even amongst officials not involved in corrupt acts. They faced reputational harm and social exclusion, reflecting how perceived corruption harmed collective wellbeing [51].

Fear of retaliation reinforced psychological harm. Transparency International documented widespread reluctance to report corruption across regions, perpetuating psychological harm and complicity [27]. Corruption also led to moral disengagement among healthcare professionals. American nurses reported moral conflict when patient care was compromised by billing practices, leading to emotional withdrawal from colleagues and patients [54]. In Afghanistan, some doctors were indifferent to corruption observed in practice, reinforcing collective cynicism [42]. Nigerian healthcare workers and Greek public sector employees described a shared sense of hopelessness in response to established corrupt practices [28,45].

#### Structural determinants of corruption.

Corruption was shown to be rooted in broader formal and informal structural determinants which shaped mental health and psychosocial effects. Resource shortages and low pay were cited as enabling conditions. In severely under-resourced health systems, corruption was described as having a functional role by enabling systems and individuals to cope with structural failure. Corruption is therefore viewed as an adaptive response to chronic constraints such as staff shortages, and weakened governance [27,29,41–44]. In Nigeria and Uganda, health workers described absenteeism and bribery as a rational response to system deficiencies such as chronic underpayment [28,44]. In Albania, patients and providers acknowledged informal payments as substitutes for wages [29].

Institutional weaknesses also reflect political and economic histories and relationships of patronage established over time. For example, the provision of food and water in Pakistan was influenced by postcolonial governance systems and landholding arrangements, and political elites retained control over essential services [53]. van Deurzen and Achim et al. hypothesised that in post-communist societies, the effect of corruption may operate differently due to unique institutional trajectories [35,36]. Both analyses were inconclusive, however they underscored the importance of understanding corruption in political transition, where weakened oversight may increase vulnerability to mental health harms [36]. Achim et al. did, however find that perceived corruption did not reduce happiness overall in LMICs, and hypothesised that collectivist norms were associated with greater wellbeing even where corruption was prevalent. They suggested the relationship between corruption and psychological outcomes is conditioned by prevailing social and cultural norms [36].

Corporate influence emerged as another structural driver. Ahmed et al. documented corporate capture in Pakistan’s nutrition sector, where formula milk companies exerted influence over health institutions despite regulations, undermining public trust and implementation of evidence-based policy [53]. Similarly, critiques of healthcare commodification in the United States highlight how market incentives prioritise profit over equity [54]. In both contexts, commercial incentives diverted priorities away from public welfare, contributing to mistrust and moral distress for service users and workers.

Ashforth and Anand offer a theoretical framework to explain how organisational structures enable corruption. In hierarchical bureaucracies with fragmented decision-making and routine-driven operations, moral accountability becomes diffused, creating “plausible deniability” across layers of leadership [47]. Legal ambiguity functions as a further rationalising resource: where laws are absent or unenforced, the resulting gap between behaviour and formal rules provides opportunities for actors to construe practices as permissible [47]. Building on this, Fleming *et al*. describe how corruption also persists through “second-order normalisation”; where it is passively tolerated by individuals and networks not directly involved, further embedding it within organisational culture [45]. Evidence from experimental research provides insight into how individual psychological processes may contribute to the structural persistence of corruption. In one experimental study, repeated acts of self‑serving dishonesty reduced amygdala responsiveness, suggesting a process of neural desensitisation that predicted the escalation of future transgressions [46]. Although this examined minor acts of deception in a controlled setting, it suggests that tolerance may be reinforced by neural desensitisation [46], in addition to the social and structural processes identified in other literature [45,47].

Informal structures were also found to perpetuate corruption through socialisation and social or cultural norms. Nigerian law enforcement officials created a “perverse pyramid of expectations”, where bribes were solicited and redistributed up to senior ranks [48]. Those who resisted were labelled “norm violators” and subject to workplace exclusion or demotion. Moral language, such as labelling non-compliers as “wicked”, functioned as a tool of social control, legitimising complicity [48]. During the pandemic, healthcare workers in HICs described social pressures to prioritise friends and family, hoarding supplies of personal protective equipment, or inappropriately prescribing treatments [27]. Corruption is also reinforced by overlapping normative systems. For example, gender norms which frame men as breadwinners, and faith norms around generosity, lead to social sanctions against those refusing to participate in corruption, making it socially costly [43]. Hierarchical structures reinforced complicity through coercion and dependency [51].

#### Individual and social protective factors for mental health and psychosocial wellbeing.

Few studies identified protective factors mitigating corruption’s psychological effects. In a study of 18 Sub-Saharan African countries, higher educational attainment, access to information, and stress-relief (measured via television ownership) were associated with lower self-reported stress [39]. These factors may reflect greater empowerment but also measure the broader construct of socioeconomic position. In Europe, depressive symptoms linked to corruption were more pronounced among individuals experiencing greater material adversity [35]. However, this relationship was not seen in longitudinal data, suggesting the protective effect of financial security may depend on other unexamined contextual factors.

DeSouza *et al.* identified several factors buffering the psychological toll among public-sector workers involved in corruption. These included having higher social standing, re-framing acts as self-preservation, and religious beliefs as sources of justification [51]. Strong faith-based ethics provided moral resilience for some health workers in Nigeria. However, these were not universally protective. Religious teachings were reinterpreted or disregarded to legitimise informal payments in life-threatening situations [43]. These findings suggest that normative beliefs affect how corruption is rationalised. They suggest that cultural and moral frameworks can both mitigate and exacerbate mental health harms.

Some professionals reported workaround measures to challenge norms. Nurses described adaptive responses and “working the system” to help uninsured patients access care. This reduced patient psychological harm, but left providers vulnerable to burnout [54]. In Nigeria, authors described how moral courage, locally referred to as “having the liver”, enabled some health managers to challenge misconduct through formal mechanisms. Formally challenging misconduct preserved integrity and reduced helplessness. However these protective effects were contingent on institutional support [28].

Awareness of entitlements within healthcare settings empowered service users to challenge informal payment demands, reducing stress and restoring agency [41,43]. Gender norms offered partial protection in some contexts. For example, female healthcare workers were perceived as less confrontational and faced lighter sanctions for non-compliance in corruption. Female service users attending clinics with male relatives were reported to have greater leverage to contest payment requests [43].

Several studies identified how social, cultural and institutional framing mitigated the psychological harms of corruption [20,29,36,43,45,48]. In Albania, informal payments were framed as expressions of gratitude or cultural obligation, maintaining a sense of dignity and mutual understanding [29]. These culturally embedded practices may serve a protective function, even as they perpetuate systemic informality. Collectivist norms in LMICs, emphasising support and interdependence, were associated with greater happiness even where corruption was prevalent [36]. At the institutional level, whistleblowing systems offer protection when effectively implemented, to encourage reporting and wellbeing [20]. However, weak governance and technology undermined these systems, leading to apathy and tolerance rather than resilience [45].

Finally, Transparency International outlined the protective role of civil society organisations (CSOs) in mitigating the harms of corruption. By documenting abuses, supporting whistleblowers, challenging social norms, and demanding accountability in healthcare, CSOs help restore trust and promote collective agency [27].

#### Mental health and psychosocial impacts of anti-corruption.

Anti-corruption efforts had mixed effects on mental health and psychosocial wellbeing. Vietnam’s 2016 anti-corruption campaign against civil servants in government and the military was associated with a 30% improvement in self-reported depression scores by the general population residing in high-corruption provinces [37]. In China, public perception of corruption improved following a national anti-corruption campaign targeting the state and enterprises, however overall mental health effects were neutral. Disaggregated analysis showed a small improvement in self-reported depressive symptoms when high‑ranking officials were investigated, suggesting visible accountability of senior officials may have benefits. However, there was no effect when lower‑ranking officials were targeted. For government employees, there was a deterioration in mental health overall. This was greatest where low-ranking officials were targeted, reflecting stressors from the workplace environment such as job insecurity and disciplinary measures [38].

Individual-level anti-corruption actions carried significant psychological costs. In a comparative study of 27 Dutch whistleblowers, 85% reported severe or very severe anxiety, depression, interpersonal sensitivity and distrust, agoraphobia symptoms or sleep problems, and 48% reached clinical levels of these specific mental health conditions [52]. Prevalence of mental health conditions was higher than among matched controls and people with work disabilities, and comparable to that among patients with cancer. Harms extended beyond employment, with negative effects reported on work, income, relationships with former colleagues, family functioning, and relationships with partners or children [52]. Greek public sector employees also described intense psychological costs, including organisational isolation and career retaliation from challenging corruption [45]. Transparency International found that 28% of individuals in Latin America reporting bribery in healthcare faced retribution, and many avoided whistleblowing for fear of being denied healthcare [27]. Ethical practice was also sanctioned in professional networks, where healthcare-workers refusing to solicit payments were labelled as “bad” colleagues and avoided, leaving them socially isolated [43]. Anticipatory fear of these outcomes may dissuade people from engaging in anti-corruption, and UNODC whistleblower protection guidelines emphasise that confidentiality and support are essential to protect wellbeing [20]. Reluctance to report was widespread [27], and those who did challenge corruption were frequently reframed within their professional networks as norm violators [43,48].

The literature described psychological processes associated with non-disclosure in greater detail than those associated with reporting. For example, rationalisation and socialisation progressively reduced ethical sensitivity, with individuals expressing doubt discouraged or sanctioned by colleagues [47]. In contrast, evidence on the factors enabling individuals to resist corruption was largely confined to service users declining demands made of them, where awareness of entitlements and greater socioeconomic resource supported refusal [41,43]. No included source examined empirically what enables individuals to report wrongdoing by others. Guidance on protective mechanisms was available [20,27], however this took the form of best-practice recommendations rather than primary research on those who had reported.

Experimental studies revealed unintended consequences of anti-corruption messaging. Descriptive norm messages framing corruption as widespread were associated with increased bribery and reduced willingness to challenge corruption [49,50]. Messaging that invoked civic duty or collective welfare was more likely to reduce bribery, but only among those with pre-existing hope or institutional trust [49]. These findings align with recommendations for norm-sensitive strategies that challenge pluralistic ignorance and reduce isolation in anti-corruption [48].

Other reports describe how interventions without systemic support could harm morale and psychosocial wellbeing. In Uganda’s health sector, frontline workers subjected to surveillance and anti-corruption raids described emotional strain and demoralisation, reporting that these interventions scapegoated staff without addressing structural drivers of corruption [44]. Such approaches undermined public trust, interpreting arrests as evidence of a dysfunctional system. In the Nigerian health sector, strong formal oversight reduced the number informal payments, but it was not eliminated. Those willing to pay ‘gifts’ continued to receive preferential treatment and information on resource availability [43]. These reforms created alternative informal economies, perpetuating corruption and its harms.

### Key informant interviews

Four semi-structured interviews were completed, lasting 40–59 minutes. Participant characteristics are summarised in Table 3. Interviewees drew on professional and research engagement with health systems rather than personal experiences of corruption.

**Table 3:** Characteristics of study participants, identifiers are anonymised.

Participant	Role	Expertise	Regional Perspective
P1	Academic Researcher	Corruption, governance and anti-corruption in health and other sectors	East Africa, Asia and Pacific
P2	Policy Advisor	Health systems and anti-corruption policy	Global
P3	Researcher at independent think tank	Political governance, corruption and social norms	West Africa
P4	Academic Researcher	Anthropology, social norms and health sector corruption	Sub-Saharan Africa

#### Individual psychological impact of corruption.

Interviewees focused on the psychological burden experienced by healthcare workers, with more limited discussion of service users. Burnout was attributed to heavy workloads and prolonged exposure to systems in which unethical practices were normalised.

This was described by one participant (Participant 2; P2) as unevenly distributed, with workers lacking patronage or influential networks more likely to be assigned to under-resourced postings. Those without connections remained in high-burden settings, contributing to helplessness and career stagnation.

Even when not directly complicit, constant anticipation of unethical demands generated vigilance and moral fatigue. As one participant explained, individuals working in highly corrupt environments must make repeated judgements about how to respond to requests for bribery or data manipulation, producing sustained psychological strain rather than episodic stress:

*“I don’t think on a daily basis about refusing to give a bribe or resisting a bribe…or what is my strategy if somebody tells me to fudge numbers…but in environments, societies where corruption is pervasive, people have to make decisions about how they will behave daily”* **P3**.

Participants also reflected on the psychological impacts of corruption for service users, particularly anxiety related to access to care. Social expectations around caregiver and gender roles were seen to intensify this, with three interviewees noting that informal payments were often framed as moral obligations. Participants described how expectations for caregiver or breadwinners shaped compliance with corrupt demands, and non-payment was framed as failure to fulfil social roles. One participant went on to describe the harmful social consequences of this:

*“People are going to make you a laughingstock, if you say the reason why your child’s [medical] condition deteriorated is because you couldn’t pay the informal payment.”* **P4**

In such contexts therefore, ethical discomfort was overridden by fear of social sanction and reputational harm. These pressures were reported to be heightened for individuals facing intersecting forms of disadvantage, including low income, limited education, rural residence, or marginalised social identities. Those positioned lower within socially determined hierarchies were described as having less autonomy to challenge demands or seek redress, leading to heightened stress and a sense of powerlessness.

Loss of agency was also described in junior healthcare workers, particularly those entering the profession through corrupt means. One participant described how debt and hierarchical systems combined to entrench dependence on corrupt practices and limit agency to challenge corruption. This dynamic was seen to be self-reinforcing, trapping individuals into a vicious cycle of corruption and psychological distress.

Overall, the interview findings provide further detail on how exposure to corruption generates cumulative psychological strain that reduces agency, and shapes responses to prioritise survival over corruption resistance.

#### Social and community-level psychological impact.

Participants described how corruption eroded trust and collective action. In contexts where corruption was normalised, relationships with colleagues and friends were marked by suspicion, particularly among those involved in anti-corruption efforts. One participant reflected on how this affected personal relationships:

*“A lot of them cut off from old friends…worried about being set up…not replying [to messages] because you don’t know who that person’s been contacted by.”* **P3**

Social hypervigilance (the heightened monitoring of others’ intentions and behaviours) undermined trust and solidarity, with isolation and withdrawal reinforcing psychological harm and limiting collective resistance.

For service users, visible breaches of fairness, such as others *“jumping the queue”* after paying informally (**P4**), damaged trust between patients and providers, as well as trust in health institutions more broadly. These experiences contributed to cynicism and disengagement from the health system. For service providers, migrant healthcare workers were highlighted as vulnerable to exploitation through informal contracts and fraudulent intermediaries. Betrayal by employers and the wider system led to a loss of professional dignity and disillusionment.

#### Structural determinants of corruption.

All participants framed corruption as a systemic outcome of institutional conditions rather than individual deviance. Poor remuneration, resource diversion, weak oversight, entrenched hierarchies, and informal networks where all cited as factors creating environments in which corruption was predictable and difficult to avoid.

Kinship obligations further shaped participation, with expectations to support family members once in a high-ranking position, encouraging engagement in corruption if formal incomes were insufficient. One participant described how non-conformity to these social expectations was sanctioned within institutional settings:

*“If you are seen to be at a variance with the norms that operate in a certain health facility or procurement committee, then there would be social sanctions… you are isolated, stigmatised, called all sorts of names because you are not aligning with the group.”* **P4**

These informal sanctions reinforced compliance and contributed to stress, anxiety, and low mood. When reinforced by multiple normative systems, such as professional, social and familial norms, corruption is more difficult to challenge, further perpetuating its effects. Professional hierarchies amplified psychological harm, particularly for junior staff unable to report misconduct without personal or professional risk.

Participants reflected on how corruption is defined and understood across contexts. One participant drew attention to the epistemological framing of corruption, such as Western-centric definitions overlooking culturally embedded forms of favouritism. They reflected on how practices such as nepotism or favour-based recruitment may be framed as professional networking rather than corruption in these contexts:

*“Nepotism is quite common and culturally acceptable, and no one says anything about [it] being corrupt…If you want to have a job, you need to have friends who introduce you to places… they put you in a course to improve your skills…mostly to improve your soft skills and networking.”* **P2**

This highlights a conceptual inconsistency in how corruption is defined, where similar practices are framed favourably in some contexts but condemned as corrupt in others. These discrepancies distort comparative understandings of corruption and its psychosocial impact across countries and regions.

Normative variation was also described within countries. One participant contrasted two districts in the same country where informal payments in health facilities carried opposite social meanings:

*“In [one district] it was...acceptable, not just common...for you to offer a bribe, informal payment...in a hospital setting...It was just part of the exchange...[in the other] it was negatively perceived...It was almost an insult. You were insulting the person by giving them something.”* ***P3***

In the setting where the practice was normalised, participants reported pressure to conform, with non-payment resulting in delayed or lower-quality care.

Political context further shaped tolerance of corruption. In settings with weak state legitimacy, corruption was described as adaptive rather than deviant. Conversely, political backing was seen as enabling reform, although participants cautioned that highly visible campaigns risked becoming performative if not accompanied by structural change.

#### Individual and social protective factors for mental health and psychosocial wellbeing.

Despite the harms of corruption, participants identified factors buffering its negative psychological impacts. These were described as helping individuals cope with morally compromised environments, although participants emphasised that some carried trade-offs which also reinforced corrupt practices.

At an individual level, participants described resilience among reformers and health workers who demonstrated a strong sense of agency, often rooted in early moral socialisation. One participant reflected on how individuals attributed resilience to formative influences and their willingness to resist social conformity, even at personal or economic costs, and how this influenced behaviour for established reformers:

*“These are individuals that have been highlighted as having a track record of integrity and anti-corruption...so each one spoke very highly of, you know, their sense of agency, autonomy…not following the crowd, not being influenced by the behaviour of others.”* **P3**

Such moral autonomy was described as fostering resistance to corruption, but it was also noted that it could generate emotional strain, particularly when ethical action resulted in isolation or informal professional sanctions.

Spiritual and religious beliefs were described as important sources of psychological stability, offering moral clarity in compromised settings. However, some participants highlighted an ambivalence in their protective role. Faith could reduce distress but could also intensify moral dissonance when ethical ideals conflicted with survival needs or be selectively reinterpreted to justify corruption.

The role of social norms in protecting mental health was similarly complex. In some contexts, fear of shame or reputational damage was described as discouraging corrupt behaviour and providing a form of social regulation. In other settings, particularly where resource scarcity was severe, corruption was reframed as necessary for maintaining services or supporting collective welfare. One participant described how such justifications were used to normalise informal charges for medications:

*“[If] there are no medicines at the facility…then they charge you a certain fee to obtain them another way, and they say, we are doing this for, actually, the greater good…people have justification for why they are charging informal payments.”* **P4**

Reciprocity and gift-giving complicated the relationship between social cohesion and corruption. In some settings, these practices were deeply embedded cultural norms that signalled respect and reinforced trust between providers and communities. Participants noted that refusing gifts could undermine relationships and negatively affect service delivery:

*“In some communities where gifts are a way of showing appreciation, where it’s so ingrained their culture, and you don’t accept it...it can have unintended negative impacts like destroying trust, which is so key for delivering services”* **P2**

Others suggested that such practices could have short-term mental health benefits by strengthening social ties, cautioning against the assumption that all informal exchanges carried psychological costs:

*“[Gifts] can have social functions as well…It may even be beneficial for mental health because you’re strengthening social ties. We shouldn’t assume it’s always coming with the mental health cost”* **P1**

Protective factors therefore operate at personal and relational levels, shaped by values, norms, and networks. While these could buffer psychological harm, they carried the risk of reinforcing corruption.

#### Mental health and psychosocial impacts of anti-corruption.

Anti-corruption efforts were described as carrying significant psychological costs for those involved in or affected by reform. These were most evident among individuals who took principled stands against corruption within unsupportive institutional environments. Interviewees described how reformers experienced chronic stress, and emotional and physical exhaustion. One participant reflected on how the aftermath of anti-corruption work resembled recovery from trauma, highlighting the cumulative and embodied nature of distress:

*“A lot had to take time off, you know, when they left that role. It was like recovering from something. I found it very interesting, it sounded very traumatic…So, really, an insight into, you know, just how psychologically, emotionally, physically, professionally tasking corruption resistance is.”* **P3**

Despite the prevalence of these negative effects, formal mechanisms for psychological support were noted to be absent. This left those engaged in anti-corruption work unprotected, and managing distress on their own.

Participants described these costs as self-reinforcing. Where sanctions for speaking out were severe, individuals became less likely to raise concerns, reducing the likelihood of disclosure and allowing corrupt practices to persist unchallenged:

*“If the sanctions are so severe for you speaking out, for you not joining the group…that is going to reduce people’s propensity, or likelihood to report corruption, to speak about it. To then become whistleblowers.”* **P4**

One participant suggested that effective whistleblower protection mechanisms would reduce this anticipatory anxiety, though noted this was inferred rather than observed.

Anti-corruption campaigns were described as having unintended psychosocial consequences. Although government-backed raids on hospitals reduced petty bribery, they also instilled fear. Healthcare workers, many of whom knew someone arrested, became anxious and distrustful. Two participants reflected on the paradox of symbolic anti-corruption campaigns which target frontline workers while ignoring elite impunity, this reinforced perceptions of injustice and deepened institutional mistrust.

Public-facing awareness and transparency campaigns were viewed as limited in their positive impact. Rather than rebuilding trust, these efforts reinforced cynicism and resignation. Participants argued that when anti-corruption initiatives are perceived as symbolic or performative, they risk functioning as forms of systemic deflection rather than meaningful reform. By failing to address underlying causes, these approaches undermine the credibility of anti-corruption initiatives and alienate workers and the public. Participants also questioned whether legal frameworks shape behaviour or experience in settings lacking the capacity to enforce them:

*“Most of the policies are more legalistic...in contexts which don’t even have effective systems for enforcement...the capacity to enforce laws is quite weak, because the informal sector is stronger than the formal sector.”* ***P4***

Therefore, without attention to the emotional toll and structural drivers of corruption, anti-corruption efforts risk causing further harm and fuelling disillusionment.

## Discussion

### Key findings

This study sought to examine how corruption in healthcare influences mental health and psychosocial wellbeing, by means of a scoping review of the literature and interviews with key experts. Corruption in healthcare emerged as systemic consequence of structural problems such as weak governance and chronic under resourcing, rather than individual rule breaking. Similar analyses in global mental health show how institutional corruption is rooted in political‑economic forces that systematically divert public sector systems from their social purpose [7,27–29,41–44,48,53]. These findings support the conceptual framework developed, whereby structural factors shape institutional and social processes which then produce psychological and psychosocial harm.

There were multiple interrelated pathways through which corruption affects mental health. Individual outcomes such as chronic stress, anxiety, hopelessness, and depressive symptoms were more pronounced in marginalised populations, reflecting intersectional disadvantage [41–43,48,53]. The interaction between systemic barriers and individual vulnerabilities align with minority stress theory, whereby distal stressors such as exclusion, and proximal stressors, such as internalised stigma, combine to produce psychological distress [56]. For those involved in corrupt practices, conflicts between ethical values and expected behaviour generated moral distress and moral injury [28,43,51,54]. Cumulative guilt and disrupted moral beliefs therefore lead to as low mood, anxiety, and maladaptive coping [57]. Refusal to participate in corrupt practices carried significant costs, such as exclusion or reputational harm, particularly in contexts where reciprocity underpins the local moral economy. As Sardan argues, such practices are embedded in systems of mutual obligation, and rejecting them may be interpreted as betrayal, resulting in informal sanctions [8]. Corruption weakened institutional and interpersonal trust, especially where individuals repeatedly encountered unfair or unpredictable treatment [29,37,40,43,53].. These patterns align with the concept of learned helplessness, which is a state in which repeated exposure to uncontrollable adverse events leads individuals to believe they cannot influence outcomes, even when opportunities for change arise. This cognitive shift reduces motivation and is associated with low mood [45,58].

The pathways described above do not operate uniformly across settings. A given act may constitute a criminal offence in one jurisdiction and remain unregulated in another, and international law distinguishes that conduct States Parties are obliged to criminalise from conduct they are required only to consider criminalising [14]. Legal status is nonetheless a poor proxy for psychological consequence. Where enforcement is weak, legal ambiguity appears to operate less as a constraint upon behaviour than as a resource for its rationalisation [47]. The mental health and psychosocial impacts of corruption therefore depend on the degree of alignment between enforcement practice and local moral meaning. Protection for reporting persons was consistently reported as a buffer against mental health harms. Therefore, where the law is the source of protection for whistleblowers, its absence could carry direct harmful psychological consequences.

Despite strong evidence that structural factors drive corruption, reform measures often paradoxically target individual behaviours. Anti‑corruption measures that increase surveillance or penalise frontline workers ignore the political economy of underfunded systems and can exacerbate psychological harm. The mixed effects of anti-corruption interventions further highlight that reform is not always protective. For example, punitive measures targeting frontline workers generated mistrust and backlash, particularly when perceived as unjust or selectively enforced [38,44,49,50]. These responses could be attributed to reactance theory, which explains that perceived threats to autonomy elicit psychological resistance and attempts to restore control [59]. For example, Uganda’s health-sector raids provoked collective protest and strikes, reflecting efforts to reassert professional dignity in the face of perceived injustice [44]. Similarly, public awareness campaigns may unintentionally normalise corruption if poorly framed, reinforcing pessimism, and creating a barrier to collective action.

Whistleblowers occupy a distinctive position within the explored evidence base. The psychological toll of corruption appears more often to suppress disclosure than to prompt it, with reluctance to report noted to be widespread [27]. Those who come forward therefore appear to act despite anticipated costs. Declining a demand carries a bounded cost, and awareness of entitlements can support refusal [41,43], whereas reporting the conduct of others extends to employment, income, family functioning and standing within professional and personal networks [48,52]. If the principal deterrent is therefore social and psychological rather than legal, protection framed solely as legal remedy addresses only part of the mechanism. van der Velden et al. argue that this imbalance between individual burden and societal benefit requires attention, both for those who have already reported and to avoid deterring future disclosure [52].

Protective factors identified were context dependent, such as strong personal and moral agency as a result of higher socioeconomic status [39]. An internal locus of control and moral agency enabled some individuals to resist corruption despite significant personal risk [60]. However, when these protective factors were undermined, resistance became psychologically costly, reinforcing withdrawal from reform. Some factors carried trade-offs. For example, gift-giving and reciprocity in some collectivist cultures could preserve social capital and dignity, whilst simultaneously entrenching informal systems. This suggests that interventions must be sensitive to local moral economies, recognising that practices labelled “corrupt” may also serve adaptive or relational functions in fragile systems.

The findings from this study extend how corruption relates to the right to health. Corruption is reported to undermine the right by diverting resources from the availability, accessibility and quality of services [23,24], a pathway also identified in the scoping review. The outcomes reported in this study, such as individual psychological harm and the psychosocial costs of disclosure, arise from the experience of corruption itself, independently of its effects on service provision. Establishing violations in specific cases would require different evidence [24], but these harms appear to fall within the scope of the right rather than outside it.

Finally, findings highlighted significant gaps in research and policy discourse. Frontline forms of corruption predominate in evidence from LMICs and structural forms in evidence from HICs, but this may reflect where research attention has been directed rather than variation in how corruption manifests, and the two cannot be distinguished from the available literature. The scoping review revealed limited healthcare-specific research linking corruption to mental health, and a reliance on cross-sectional designs and self-reported measures. Larger studies in the scoping review examined corruption across public services rather than within healthcare and relied upon perceived rather than experienced corruption, whilst healthcare-specific studies were predominantly smaller and qualitative. Causal inference regarding the mental health consequences of healthcare corruption specifically is therefore limited by the evidence currently available. Methodologically, there is a gap in ethnographic and longitudinal research to capture the complex, and long-term interactions between corruption and mental health. Without this, measurement tools may fail to operationalise or adequately assess the extent of corruption’s mental health outcomes. The low engagement of potential key informants may reflect a lack of cross‑sector awareness. Mental health experts may not yet recognise corruption as relevant, while governance specialists may overlook mental health outcomes. This reinforces the need for interdisciplinary research and conceptual integration.

### A new conceptual framework

The conceptual framework developed in this analysis represents a novel contribution. It synthesises findings from this report into a model linking determinants of corruption, negative mental health and psychosocial outcomes, and the unintended effects of anticorruption efforts (Fig 2). In line with models from Glynn and Vian, corrupt acts are framed as symptoms of deeper system conditions that are distributed across macro, meso, and micro levels [4,10].

**Fig 2:**
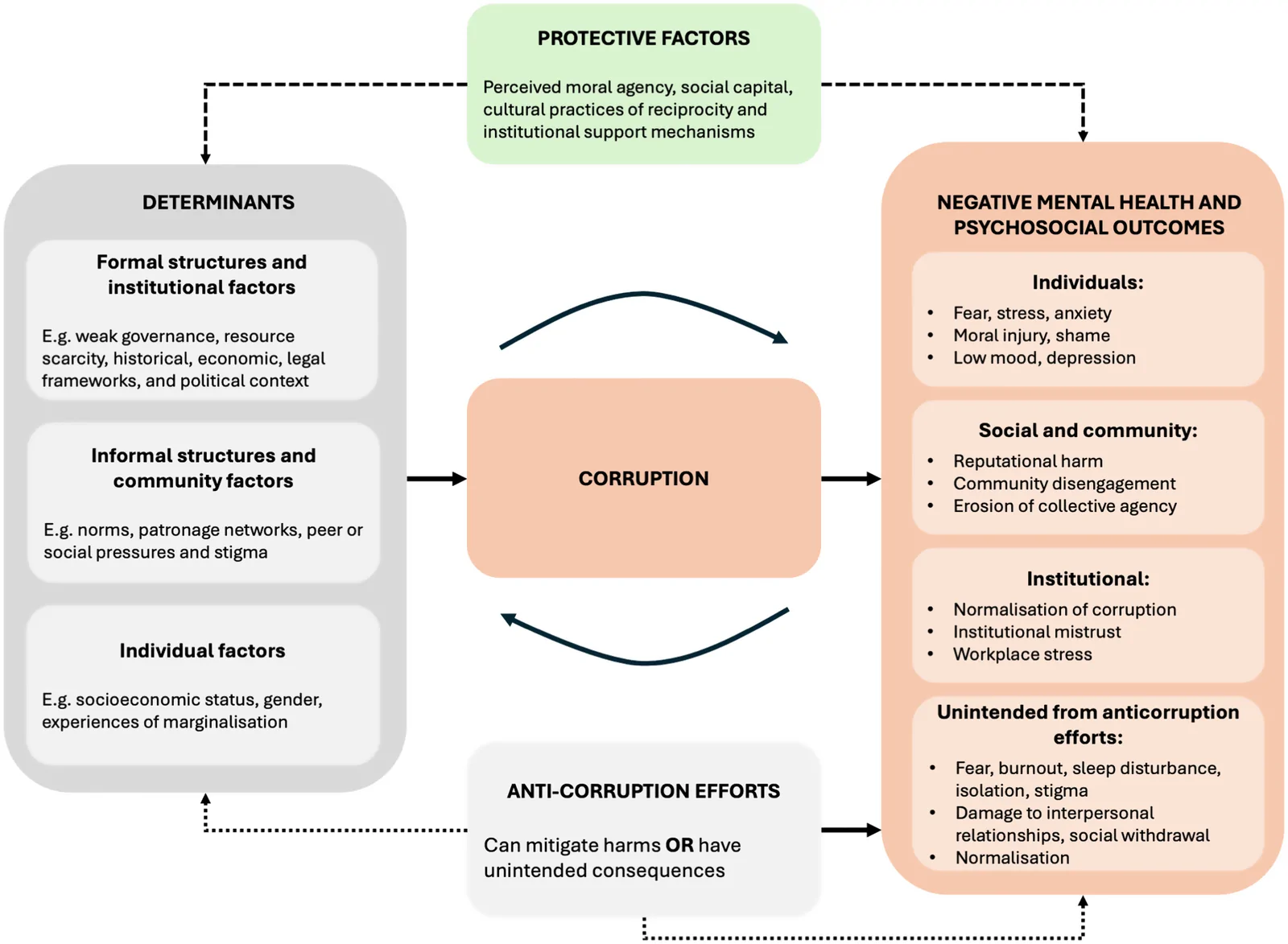
Conceptual framework illustrating how structural, informal, social, and individual determinants contribute to corruption and its mental health and psychosocial impacts. It also shows potential unintended consequences of anti-corruption efforts, feedback loops sustaining corruption over time, and protective factors acting as moderators that buffer harmful effects.

The framework illustrates three types of determinants found in this report. These include structural determinants such as weak governance and chronic underfunding of services; informal and community-level factors such as entrenched norms, and peer or social pressures; and individual determinants including gender, socioeconomic status, and experiences of marginalisation. These determinants interact to create the conditions where corruption is normalised and sustained, and reflect the social determinants of health [34].

Four categories of negative psychosocial and mental health outcomes were identified from findings, which appeared to be structurally determined and differentially distributed across populations [28,29,42,45,54]. For individuals, outcomes include stress, anxiety, low mood, and moral injury. Social and community harms include reputational harm, community disengagement and erosion of collective agency. Institutional negative outcomes include mistrust and normalisation of corruption. Anti-corruption efforts may have unintended consequences such as fear, damaged interpersonal relationships, and inadvertent normalisation of corruption. These perpetuate corruption, alongside the feedback loops that are shown between determinants and outcomes.

Protective factors were reported to operate alongside categories of negative outcomes [20,35–37,39,45]. For individuals, a perceived sense of moral agency, stemming from education, role models, socioeconomic status, or faith, may be protective. However, their effects are context dependent. At the social and community level, social capital, community practices of reciprocity, and the role of CSOs offer protection by maintaining trust and collective agency. Within institutions, robust reporting systems buffer negative outcomes. Protective factors in anti-corruption reform require further investigation but may include an approach which addresses structural drivers of corruption, and access to organisational and psychosocial support for reformers.

Individuals and communities have little choice to act on corruption given the complex environment of underfunding, acceptability and norms favouring the status-quo. Ability and willingness to engage in productive action is weakened by distress, withdrawal, and cynicism. The framework is designed to demonstrate these outcomes and locate policy leverage points across levels, combining reforms addressing root causes with strategies that mitigate harm and support those exposed to corruption.

### Limitations

This study combined a scoping review and key informant interviews to synthesise evidence across diverse contexts. Triangulation strengthened the conceptual framework, clarifying pathways from corruption to mental ill-health and potential protective factors. As a scoping review, the study aimed to map the emerging but limited evidence base, rather than undertake formal critical appraisal. Most quantitative studies were cross-sectional, used self-reported outcomes, and heterogeneous measures of corruption and mental health outcomes. This limits causal inference. The quality and regional coverage of grey literature also varied.

Another limitation was the restriction of the review to English-language sources and databases which predominantly index English-language journals, which will have introduced geographic bias. Included evidence covered Sub-Saharan Africa (Ghana, Nigeria and Uganda), Europe, Asia and North America. This restriction may have also excluded forms of corruption described using local terminology, and conceptualisation of corruption and mental health outcomes prevalent in English-language literature. Key informant interviews were conducted in English, which may have favoured perspectives from international policy and research networks over those of local healthcare practitioners or service users. As a result, the findings and conceptual framework predominantly reflect findings from the available contexts and perspectives.

Healthcare-specific evidence was limited, and the review incorporated findings from other settings where mechanisms were judged to be relevant. While this strengthened conceptual insight, it may limit the transferability of some findings to specific healthcare contexts, particularly given the variation in health systems and governance globally.

Time and resource constraints restricted review depth, and analysis was conducted by one reviewer. The small number of expert interviews reflects the exploratory nature of this topic, and the limited pool of specialists working at the intersection of corruption and mental health. Interviews were conducted within a short timeframe, which may have excluded some perspectives.

A key limitation concerns both the definition and the distribution of corruption examined. The Transparency International definition was taken as a reference point to compare mechanisms across setting, rather than determine definitively which practices should be classified as corrupt. Included sources did not share a common definition or operationalisation, limiting direct comparison. Prevailing definitions of corruption have been shaped predominantly by an international policy tradition situated within high-income and donor institutions [6]. Applying an externally derived definition therefore risks classifying as corrupt practices that carry different moral meaning in context, which may itself shape the psychological consequences reported. The various forms of corruption reported in this study were concentrated on frontline corruption, particularly informal payments, absenteeism and bribery in service delivery, with little evidence on procurement fraud, regulatory capture, high-level conflicts of interest or state capture. This mirrors a documented imbalance in the wider health sector corruption literature, in which higher-level forms receive comparatively little empirical attention despite their substantial system-wide impact [5]. The included evidence likewise rarely addressed the legal treatment or outcomes of corruption cases, and their effect on mental health. Legal frameworks could therefore be mapped as a structural determinant but not characterised in depth, and comparative evidence across settings with differing legal treatment of the same practice should be further explored.

Conceptual variability in defining mental health outcomes further limited synthesis. Mechanisms identified in one setting may not generalise to others, and few studies used validated psychological measures or disaggregated findings by demographic characteristics. Moreover, it remains unclear how corruption-driven mental distress reported qualitatively differs from clinically defined conditions, such as anxiety or depression.

### Recommendations

We suggest a range of recommendations for research, policy and practice, which should be refined through further empirical study given the exploratory nature of this study. Three principles cut across stakeholder groups. First, corruption should be recognised as a public mental health issue alongside its governance and economic dimensions, so that reforms address both structural drivers and their psychological consequences. Second, because these harms arise from intersecting determinants spanning governance, institutions, workforce and community, they are unlikely to be addressed through isolated interventions. Third, a rights-based approach to health offers an existing framework for anti-corruption efforts. Integrating the right to health into anti-corruption reform and policy has been proposed as a means of strengthening such measures and would extend oversight to psychological as well as material harm.

For policymakers, the primary recommendation is that psychological harm should be considered as an outcome of corruption rather than an incidental effect. Mental health indicators should be incorporated into the monitoring and evaluation of anti-corruption reforms, and likely psychological consequences assessed prospectively, particularly if enforcement targets frontline workers. System‑wide reforms, including measures to address mental health consequences, must be designed with sensitivity to local political economy and context of healthcare provision.

For health system managers and employers, the costs of corruption were uneven within institutions, concentrating on those with least power. Confidential reporting routes that do not depend upon line management and supportive supervision are recommended. Moral distress and moral injury warrant recognition as occupational risks within staff wellbeing provision, rather than as individual vulnerability.

For healthcare professionals and professional bodies, corruption awareness and mental health literacy should be embedded within training and continuing professional development, supported by peer support structures for staff navigating ethical pressure. Professional guidance should recognise moral injury arising from systemic constraint as distinct from individual misconduct.

For community organisations and service users, awareness of entitlements emerged as protective, enabling service users to challenge demands for informal payment and restoring a sense of agency. Initiatives supporting such awareness, alongside confidential reporting channels accessible to service users and community-led accountability mechanisms developed in partnership with civil society are therefore recommended. Public communication should explicitly link corruption to health outcomes and promote collective responsibility. Messaging should avoid placing responsibility for refusal upon individuals without acknowledging the costs that refusal carries.

For oversight bodies and those engaged in anti-corruption reform, including whistleblowers, statutory protection and independent oversight are required to reduce professional and psychological risk. Protection mechanisms should attend to psychological safety and sustained support, beyond legal redress, given the breadth and persistence of the harms reported. Services such as occupational health represent an existing point of contact which could screen for disclosure-related distress where circumstances are known.

For researchers and research funders, the evidence base requires expansion through larger-scale longitudinal, ethnographic and healthcare-specific studies, with attention to protective factors and differential impacts across population subgroups and experiences of corruption rather than perceived corruption. Future research should examine forms of corruption beyond individual acts, such as higher-level forms of corruption, and their determinants. It should also draw on non-English sources and regional databases across a wider range of contexts. This will help map the impact of a broader range of corruption forms on mental health and psychosocial outcomes. Comparative studies across settings with differing legal and normative treatment of the same practice would establish whether context modifies psychological outcomes. The mental health consequences of anti-corruption interventions and reform should be assessed prospectively to safeguard against unintended harm and explore factors which enable reporting.

## Conclusion

This study examined how corruption in healthcare intersects with mental health, drawing on a scoping review and key informant interviews. Findings suggest that corruption functions as an under-recognised social determinant of mental health, while anti-corruption efforts can carry both protective and harmful psychosocial effects. As noted in the limitations, the evidence reviewed was concentrated on frontline forms of corruption and predominantly reflected definitions centred on individual corrupt acts, published in English. The mental health effects of higher-level corruption and its structural determinants therefore require further investigation, and the mental health burden and psychosocial harms associated with corruption described in this study is likely to be underestimated. Integrating mental health considerations into anti-corruption research and policy is essential for advancing equitable and resilient health systems.

## Supporting information

S1 File PRISMA-ScR Checklist. Completed Preferred Reporting Items for Systematic reviews and Meta-Analyses extension for Scoping Reviews (PRISMA-ScR) checklist, with the page number where each item is reported.
(DOCX)

S1 Text Search strategies. Search strategies used for MEDLINE, adapted for Global Health, APA PsycINFO, Scopus, and Google.
(DOCX)

S2 Text Key informant interview topic guide. Semi-structured topic guide used for the virtual key informant interviews.
(DOCX)

S3 Text Participant information sheet. Information sheet given to key informants before they consented to interview.
(DOCX)
