# Supplementary material for: The hidden mental health burden of corruption in healthcare: Findings from a scoping review and key informant interviews
Source: PLOS Glob Public Health. 2026 Oct 30;6(10):e0007509. doi: 10.1371/journal.pgph.0007509 (PMC13722154; doi:10.1371/journal.pgph.0007509)
Supplement: Supplementary file 2 [file pgph.0007509.s002.docx]

## A. Database Search Strategy

Search strategies were adapted for databases (MEDLINE, Global Health, APA PsycINFO and SCOPUS). Searches were approximated to the MEDLINE search which is listed below with search terms and appropriate Medical Subject Headings:

1 (corrupt* or bribe* or (misus* adj2 funds) or fraud or "institutional failure" or "anti-corruption" or "informal payment*" or governance or transparen* or accountability).mp.
2 ((mental adj2 (health or well-being)) or (psychological adj2 (impact or effect or well-being or distress or burden)) or (psychosocial adj2 (impact or effect or well-being or distress or burden)) or stress or burn-out or anxiety or "moral injury" or depression or trauma* or normali#ation or morale or "post-traumatic stress disorder").mp.
3 "behavior and behavior mechanisms"/ or mental disorders/
4 (actor* or patient* or "service-user*" or client* or "health-care worker*" or manager* or professional* or whistleblower* or staff or reformer* or provid#r* or advocate* or activist* or personnel).mp.
5 health occupations/ or crime victims/ or "emigrants and immigrants"/ or friends/ or "health disparate minority and vulnerable populations"/ or legal guardians/ or medically uninsured/ or parents/ or patients/ or research subjects/ or survivors/ or tissue donors/ or visitors to patients/ or volunteers/
6 ("public sector" or healthcare or hospital or organi#ation or institution* or "health system" or "health-care system" or "public service*" or system* or government*).mp.
7 informal sector/ or public sector/
8 2 or 3
9 4 or 5
10 6 or 7
11 1 and 8 and 9 and 10
12 limit 11 to english language
13 limit 12 to yr="2000 -Current"

## B. Google Search Strategy

On 18th June 2025:

(Corruption OR Informal Payment OR Misuse Of Funds OR Fraud OR Anti-corruption OR Whistleblowing OR Anticorruption) AND (Mental health OR Psychological OR Psychosocial) AND (Health System OR Public Sector OR Organisation OR Organization OR Institution)
