# Supplementary material for: The hidden mental health burden of corruption in healthcare: Findings from a scoping review and key informant interviews
Source: PLOS Glob Public Health. 2026 Oct 30;6(10):e0007509. doi: 10.1371/journal.pgph.0007509 (PMC13722154; doi:10.1371/journal.pgph.0007509)
Supplement: Supplementary file 3 [file pgph.0007509.s003.docx]

**Topic Guide**

**Project Title:**

**Beneath the Surface: Exploring Mental Health Consequences of Corruption in Healthcare.**

**A Health Policy Report**

**Estimated Duration: 45–60 minutes**

**Mode: Virtual (Zoom or equivalent)**

**Consent: Audio-recorded and transcribed with written consent**

**Section A: Introduction & Consent (5 min)**

Brief intro to the project: exploring how corruption in healthcare affects mental health.

Confirm consent for participation and audio recording.

Remind participants of anonymity and voluntary nature of the interview.

**Section B: Background and Role (5–10 min)**

Can you briefly describe your current role and background?

How have you been involved with work around corruption in healthcare systems, mental health, or anti-corruption efforts?

**Section C: Experiences of Corruption and Psychosocial Effects (15–20 min)**

In your experience or observation, what forms of corruption are most prevalent in healthcare systems?

How have these forms of corruption affected different actors (patients, providers, administrators)?

Follow-ups:

- Emotional or psychological toll for service users? (e.g. stress, shame, distrust)
- Mental health impact on healthcare providers (e.g. burnout, moral injury)?
- How is corruption discussed or experienced as part of the workplace culture?

**Section D: Reformers and Mental Health Burden (10–15 min)**

For individuals involved in anti-corruption work (e.g. whistleblowers, reformers), what kinds of psychological or emotional burdens are common?

Follow-ups:

- Retaliation, institutional pressure, isolation, fear?
- Are there formal or informal support systems available to them?
- How do people cope in contexts where corruption is systemic or normalised?

**Section E: Structural and Sociopolitical Influences (10 min)**

What structural or institutional factors influence how corruption affects mental health?

Prompts:

- Power dynamics, regulatory failure, inequality, weak accountability?
- How do cultural or political norms shape people’s responses to corruption?

**Section F: Policy & Conceptual Reflections (10–15 min)**

From your perspective, what types of policy responses could reduce psychological harm linked to corruption and anti-corruption efforts?

Prompts:

- Mental health support?
- Whistleblower protections?
- Transparency or accountability mechanisms?

What support would help individuals and institutions to engage in anti-corruption efforts more safely and sustainably?

What do you think are the key pathways through which corruption affects mental health?

(If time allows):

What does "collective action" look like in contexts of entrenched corruption? Can it help reduce psychological burden or build resilience?

**Section G: Final Reflections (5 min)**

Is there anything else you think is important to mention that we haven’t covered?

Are there other people or organisations you think I should speak with?
