# Supplementary material for: The hidden mental health burden of corruption in healthcare: Findings from a scoping review and key informant interviews
Source: PLOS Glob Public Health. 2026 Oct 30;6(10):e0007509. doi: 10.1371/journal.pgph.0007509 (PMC13722154; doi:10.1371/journal.pgph.0007509)
Supplement: Supplementary file 4 [file pgph.0007509.s004.docx]

**Participant Information Sheet**

**Title of Project:**

Beneath the Surface: Exploring Mental Health Consequences of Corruption in Healthcare, A Health Policy Report

**Introduction**

*We would like to invite you to take part in interviews for a MSc Summer Project. Participating in interviews is entirely up to you. Before you decide, you need to understand why the project is being done and what it would involve. The MSc candidate completing the project will go through this information sheet with you, and answer any questions you may have. Ask questions if anything you read is not clear or you would like more information. Please feel free to talk to others about the project if you wish. Take time to decide whether or not to take part*.

**What is the purpose of the study?**

*A London School of Hygiene and Tropical Medicine (LSHTM) Masters student is undertaking a project to explore how corruption in healthcare systems affects mental health—for those accessing healthcare, delivering services, and for people trying to challenge corruption (e.g., whistleblowers or anti-corruption reformers). Your insights will help us better understand these links and make recommendations to reduce harm and support those affected.*

**Why have I been asked to take part?**

*You have been invited because you are a key informant with expertise or experience relevant to corruption and/or mental health in healthcare settings. This may be through professional roles in policy, governance, mental health, social science, or anti-corruption initiatives.*

**Do I have to take part?**

*No. Participation is entirely voluntary. You may withdraw at any time without giving a reason. If you choose to withdraw, we will not collect any information, and you can ask us to delete previously collected information.*

**What will I have to do?**

*You will be invited to take part in a one-to-one virtual interview lasting approximately 45–60 minutes. The interview will explore your insights and experiences related to the impact of corruption in healthcare systems. Specifically, we will discuss:*

- *Observations or experiences of corruption and how they affect different individuals, including patients, healthcare workers, and reformers*
- *Emotional, psychological, or mental health impacts linked to both corruption and anti-corruption efforts*
- *The broader institutional, structural, or sociopolitical factors that shape these effects*
- *Your views on potential policy responses, support mechanisms, and pathways for change*

*With your consent, the interview will be audio recorded and transcribed for analysis. All data will be anonymised, and any identifiable information will be removed from transcripts and reports.*

**What are the possible risks and disadvantages?**

*Some questions may involve sensitive topics such as stress or difficult professional experiences. You may choose not to answer any question or end the interview at any point. If the conversation causes distress, you can pause or stop the interview.*

**What are the possible benefits?**

*We cannot promise the study will help you but the information we get from the study will help our knowledge and understanding of this research area in Anti-Corruption in healthcare settings.*

**What if something goes wrong?**

*If you have a concern about any aspect of this study, you should ask to speak to the MSc candidate completing the project who will do their best to answer your questions:* [*madura.nanthakumaran1@student.lshtm.ac.uk*](mailto:madura.nanthakumaran1@student.lshtm.ac.uk)*. If you remain unhappy and wish to complain formally, you can do this by contacting The Research Governance and Integrity Office at* [*rgio@lshtm.ac.uk*](mailto:rgio@lshtm.ac.uk) *or +44 (0) 20 7927 2626.*

*The London School of Hygiene and Tropical Medicine holds insurance policies which apply to this project. If you experience harm or injury as a result of taking part in this study, you may be eligible to claim compensation.*

**Can I change my mind about taking part?**

*Yes. You can withdraw from the project at any time. You just need to tell the MSc candidate you no longer wish to take part. If you withdraw from the study we will destroy all your tape recorded interviews.*

**What will happen to information collected about me?**

*All the information we collect during the course of the research will be kept confidential and there are strict laws which safeguard your privacy at every stage.*

*The London School of Hygiene and Tropical Medicine are based in the United Kingdom. To follow the United Kingdom’s data protection regulations, we must inform you of how we will use and store your personal data.*

*As a university, we use personally identifiable information to conduct research to improve health, care and services. As a publicly funded organisation, we have to ensure that it is in the public interest when we use personally identifiable information from people who have agreed to take part in research. This means that when you agree to take part in a research study, we will use information collected from you to conduct and analyse the project.*

*Only the project staff and authorities who check that the study is being carried out properly will be allowed to look at information about you. Information will include your initials, contact details and roles used for project to be listed. We will keep all information about you safe and secure.*

*Your personal details, meaning your name and other identifiable information, will be kept in a different safe place to the other study information and will be destroyed at the end of the project.*

*At the end of the project, the study data will be archived at London School of Hygience and Tropical Medicine MSc project databases. Your personal information will not be included and there is no way that you can be identified.*

*The London School of Hygiene and Tropical Medicine will act as the data controller for this study. This means that they are responsible for looking after your information and using it properly.*

**What are your choices about how your information is used?**

*You can stop being part of the project interviews at any time, without giving a reason, and we will destroy the information collected through interviews.*

**Where can you find out more about how your information is used?**

*You can find out more about how we use your information*

- *At* [*https://www.lshtm.ac.uk/files/research-participant-privacy-notice.pdf*](https://www.lshtm.ac.uk/files/research-participant-privacy-notice.pdf)
- *by asking one of the research team*

**What will happen to the results of this study?**

*Findings will be shared through an MSc dissertation and may be published in academic journals or policy reports. You will not be identifiable in any materials. If you would like to receive a summary of the findings, you can let us know.*

**Who is organising and funding this study?**

*This study is being conducted as part of an MSc in Public Health at the London School of Hygiene & Tropical Medicine. There is not additional funding for completion of this project. The London School of Hygiene & Tropical Medicine have full responsibility for the project including the collection, storage and analysis of your data, and will act as the Data Controller for the study. This means that we are responsible for looking after your information and using it properly.*

**Who has reviewed this study?**

*All research involving human participants is looked at by an independent group of people, called a Research Ethics Committee, to protect your interests. This study has been reviewed and given favourable opinion by The London School of Hygiene and Tropical Medicine Research Ethics Committee (ref:* *31966).*

**Further information and contact details**

Thank you for taking time to read this information sheet. If you think you will take part in the study please read and sign the consent form.

If you would like any further information, please contact Dr Madura Nanthakumaran who can answer any questions you may have about the study.

Contact details:

[madura.nanthakumaran1@student.lshtm.ac.uk](mailto:madura.nanthakumaran1@student.lshtm.ac.uk)

**Consent Form**

Study Title: *Beneath the Surface: Exploring Mental Health Consequences of Corruption in Healthcare*

Thank you for agreeing to take part in this interview. The interview will last around 45–60 minutes and will be audio recorded and transcribes to ensure accuracy. All information you provide will be kept confidential and fully anonymised. The aims of the project will be explained to you, and you’ll have the opportunity to ask any questions before we begin. Your insights will help inform future health policy on the mental health impacts of corruption in healthcare.

Please read and initial each statement:

- I confirm I have read and understood the Participant Information Sheet.
- I understand that participation is voluntary and I can withdraw at any time.
- I give permission for the interview to be audio recorded and transcribed.
- I understand my responses will be anonymised and kept confidential.
- I understand that anonymised quotes may be used in reports or publications.
- I consent to the use of my data for this research project.

☐ I would like to receive a summary of the project findings.

| Email | ……………………………………………………………………………………………… |
| --- | --- |
| Telephone | ……………………………………………………………………………………………… |
| Name of participant | ……………………………………………………………………………………………… |
| Signature of participant | ……………………………………………………………………………………………… |
| Date | ……………………………………………………………………………………………… |
| Name of facilitator | ……………………………………………………………………………………………… |
| Signature of facilitator | ……………………………………………………………………………………………… |
| Date | ……………………………………………………………………………………………… |
